# Sentinel lymph node detection using magnetic resonance lymphography with conventional gadolinium contrast agent in breast cancer: a preliminary clinical study

**DOI:** 10.1186/s12885-015-1255-4

**Published:** 2015-04-02

**Authors:** Chuanming Li, Shan Meng, Xinhua Yang, Daiquan Zhou, Jian Wang, Jiani Hu

**Affiliations:** 1 Department of Radiology, Southwest Hospital, Third Military Medical University, 30 Gaotanyan Road, Chongqing, 400038 China; 2 Department of Breast Surgery, Southwest Hospital, Third Military Medical University, 30 Gaotanyan Road, Chongqing, 400038 China; 3 Department of Radiology, Wayne State University, Detroit, MI 48331 USA

**Keywords:** Breast cancer, Lymph node, Metastasis, Magnetic resonance lymphangiography, Gadolinium

## Abstract

**Background:**

Sentinel lymph node (SLN) mapping is the standard method for axillary lymph node staging in patients with breast cancer. Blue dye and radioisotopes are commonly used agents to localize SLNs, but both have several disadvantages. The purpose of this study was to evaluate magnetic resonance lymphography with a gadolinium-based contrast agent (Gd-MRL) in sentinel lymph node identification and metastasis detection in patients with breast cancer.

**Methods:**

Sixty patients (mean age: 46.2 ± 8.8 years) with stage T1- 2 breast cancer and clinically negative axillary lymph nodes participated in this study. After 0.9 ml of contrast material and 0.1 ml of mepivacaine hydrochloride 1% were mixed and injected intradermally into the upper-outer periareolar areas, axillary lymph flow was tracked and sentinel lymph nodes were identified by Gd-MRL. After SLN biopsy and/or surgery, the efficacy of SLN identification and metastasis detection of Gd-MRL were examined.

**Results:**

Ninety-six lymph nodes were identified by Gd-MRL as SLNs (M-SLN), and 135 lymph nodes were detected by blue dye-guided methods as SLNs (D-SLN). There was a strong correlation (P < 0.001) between the SLN numbers found by these two methods. Using blue dye-guided methods as the gold standard, the sensitivity of Gd-MRL was 95.65% and the false-negative rate was 4.3% for axillary lymphatic metastasis detection. With heterogeneous enhancement and enhancement defect as the diagnostic criteria, Gd-MRL gave a sensitivity of 89.29% and specificity of 89.66% in discriminating malignant from benign SLNs.

**Conclusion:**

Gd-MRL offers a new method for SLN identification and metastasis detection in patients with breast cancer.

## Background

Breast cancer is the second leading cause of death from cancer, with more than 200,000 new cases diagnosed each year in the United States [1]. The regional spread of tumor cells from the breast primary lesion to the axillary lymph nodes is a well-recognized step in the metastatic process for breast cancer [2]. Therefore, accurate detection of axillary lymph node metastases is critical for surgical planning, adjuvant therapy planning, and prognostication.

Histopathological examination of sentinel lymph node biopsy (SLNB) is the standard procedure in the determination of axillary lymph node status [3,4]. Radioisotopes (such as technetium-99 m sulfur colloid and technetium-99 m albumin) and blue dyes (such as isosulfan blue or patent blue) are widely utilized as lymphatic mapping agents. However, the use of radioisotopes is associated with radiation exposure/safety issues for the patient, surgeon, pathologist, and other medical staff, and there may be limited availability of radioisotope (technetium-99 m) and gamma detection probe equipment at some hospitals that do not have nuclear medicine capabilities [5,6]. Blue dyes are inexpensive and relatively easy to use for intraoperative lymphatic mapping. However, intraoperative lymphatic mapping with blue dyes can be associated with allergic/anaphylactic reactions, and lacks the ability to visualize the pre-incision anatomical relationship between tumor, lymph vessels, and SLNs, thus limiting the surgeon’s ability to decide upon exact placement of the surgical incision [7-9]. Therefore, a safe, simple and non-invasive preoperative method is needed in clinical practice.

Magnetic resonance lymphography (MRL) is a technique that employs magnetic resonance imaging after interstitial injection of a contrast agent [10-15]. In a past study, we have established an effective MRL protocol with gadolinium (Gd)-based contrast agents (Gd-MRL) that can generate high-resolution images of axillary lymphatic vessels and nodes [16]. The purpose of this study was to evaluate Gd-MRL in sentinel lymph node (SLN) identification and metastasis detection in patients with newly diagnosed breast cancer.

## Methods

### Ethics statement

All research procedures were approved by the ethics commission of Southwest Hospital of China and were conducted in accordance with the Declaration of Helsinki. Written informed consent was obtained for all patients.

### Patients

From January 2012 to Oct 2013, a total of 68 consecutive patients with stage T1- 2 breast cancer and clinically negative axillary lymph nodes who underwent sentinel lymph node biopsy were enrolled in this study. Patients with multiple primary tumors, prior axillary surgery, preoperative chemotherapy, or who were pregnant were excluded. Patients with a contraindication to MR imaging or a known allergy to the contrast agents were also excluded. The study population comprised 60 patients (age ranging from 31 years to 62 years; mean age: 46.2 ± 8.8 years), including 47 with invasive ductal carcinoma, 10 with invasive lobular carcinoma, 2 with tubular carcinoma and 1 with medullary carcinoma.

### Contrast agent and administration

Gadopentetate dimeglumine (GD-DPTA) (Magnevist, Bayer Schering Pharma AG, Berlin, Germen) with a gadolinium (Gd) concentration of 0.5 mol/L was used for contrast. A 1-ml tuberculin syringe and 26-gauge needle were used for Gd-DPTA injection. A total of 0.9 ml contrast material and 0.1 ml mepivacaine hydrochloride 1% were mixed and injected intradermally into the upper-outer periareolar areas [17]. Mepivacaine hydrochloride was added to alleviate pain during intradermal injection. The injection site was massaged gently for 90 seconds to promote migration.

### MRI

MR imaging for all subjects was performed on a 3.0 T whole-body system (Magnetom Trio, Siemens Healthcare, Erlangen, Germany) with a 12-channel matrix body coil. Patients were placed in the supine position with their arms elevated, similar to the position used during surgery. The conventional imaging protocol consisted of an axial T1-weighted fast spin echo (T1-FSE) sequence, an axial diffusion-weighted sequence (TR/TE 6548/65 ms; FOV 340 × 340 mm^2^; b values of 50, 200 and 500 sec/mm^2^), and an axial T2-weighted fat-suppressed sequence (TR/TE 4000/70 ms; inversion delay 125 ms; flip angle 90°; FOV 340× 340 mm^2^). For Gd- MRL, 3D fast spoiled gradient-recalled echo T1-weighted images with fat saturation (volumetric interpolated breath-hold examination, VIBE) were acquired prior to the administration of Gd-DTPA with the following parameters: TR/TE = 8.0/3.9, flip angle 15°, FOV = 340 mm × 340 mm, acquisition matrix = 512 × 512, slice thickness = 1 mm. After intradermal administration of the contrast material, the same imaging sequence (VIBE) was repeated at 9, 12, 15, 18, 21 and 24 minutes. Maximum-intensity projections were used to improve visualization of lymphatic vessels. Finally, a bolus intravenous injection of 0.1 mmol/kg gadopentetate dimeglumine followed by a 20 ml saline flush at an injection rate of 2 ml/s was administered, and the sequence was repeated.

### SLN identification and skin marking

During scanning, lymph vessels from the injection site to the axilla were stained with Gd-MRL. For Gd-MRL, the SLN was defined as the first lymph node visualized on the lymph vessel draining directly from the injection site (M-SLN) . In some patients, more than one lymphatic vessel drained directly from the injection site. In these patients, the first visualized lymph node along each lymphatic vessel draining directly from the injection site was considered a sentinel node (M-SLN). [17]. The marking of the M-SLN spot was performed using a skin-marker method [18,19]. A cod liver oil capsule, which is usually used for MRI localization, was first attached to the skin. After 3D Gd-MRL images were reconstructed at each time point with maximum-intensity projection and surface-rendering techniques, the distance and angle between the marker and the M-SLN were analyzed, and the marker was adjusted appropriately. Usually, 2–3 scans were needed to get an accurate correspondence between the M-SLN and the skin oil marker. Finally, the M-SLN location was marked on the skin surface using an oil painting pen.

### SLNB and histopathologic analysis

Sentinel lymph node biopsy was performed for all patients. After the induction of general anesthesia, a subareolar injection of 3 ml methylene blue was performed, and the injection site was massaged gently for 90 seconds to promote migration using the same technique as for MR lymphography. M-SLNs located just under the marking site determined by MR lymphography were removed first. If several nodes lay close to others, they were discriminated by size and morphological character. Then, other SLNs stained by methylene blue were detected and excised by following the blue lymphatic vessels. These were designated as D-SLN. All dissected M-SLNs and their MRI images were examined to confirm they were identical or closely similar in shape and size. All of the resected LNs were fixed in formalin, 2-mm serial sections were prepared, and histopathologic evaluations were made for the presence of cancer metastasis. If no SLN metastases were present, LN dissection was not performed, but when there were metastases in resected SLNs, it was.

### Data analysis

Two radiologists with 10 and 12 years of experience in breast imaging analyzed the images prospectively. Correlations between the number of SLNs detected by Gd-MRL and the blue dye-guided method were analyzed. Heterogeneous enhancement and enhancement defect are characters of metastatic nodes in Gd-MRL, as shown by our past study [16]. According to these criteria, the SLN metastasis diagnostic ability, including sensitivity and specificity of Gd-MRL, were calculated. All statistics were computed using SPSS statistical software (version 16.0, SPSS Inc., Chicago, Illinois). P values of 0.05 were considered statistically significant.

## Results

All breast cancer patients completed their examinations successfully. Six showed swelling at the site of contrast injection, and all of them disappeared within approximately 30 minutes. There was no allergic or other acute reaction.

**Figure 1:**
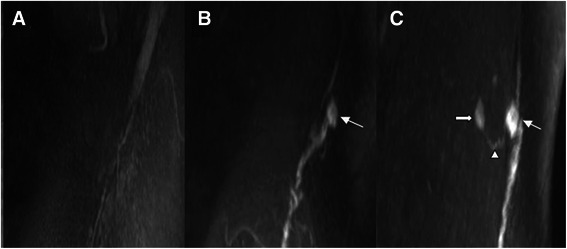
**Gd-MRL images in a 46-year-old patient with left breast ductal carcinoma.** Compared with pre-contrast images **(A)**, the axillary lymphatic pathway was dynamically stained 9 min **(B)** and 18 min after contrast injection **(C)**. The SLN could be easily identified on Gd-MRL (white thin arrow). One distant node (white thick arrow) and its connection lymph vessel (white triangle) with an SLN are also displayed.

Sentinel lymph nodes could not be delineated on pre-injection MR imaging (Figure 1 A). After injection of Gd-DTPA into the subareolar breast tissue, the dynamic multiple-angle views of the 3D Gd-MRL image showed the axillary lymph flowing into the SLN (Figure 1 B, C). The SLN could be identified easily on Gd-MRL. Distant nodes and their connection lymph vessel with SLNs were also displayed (Figure 1 C).

**Figure 2:**
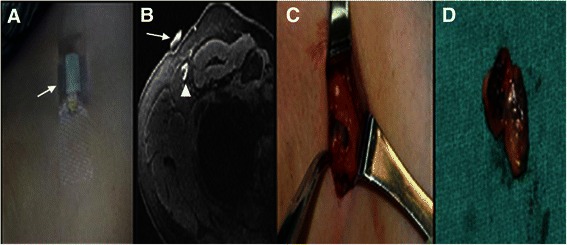
**In a 42-year-old patient with right breast ductal carcinoma. A**: The skin marker of a cod liver oil capsule (white arrow) was attached to the skin. **B**: The skin marker (white arrow) correlated well with the target lymph node (white triangle). **C, D**: During operation, the lymph node was easily resected under the guidance of the skin marker.

In total, 96 lymph nodes were identified by Gd-MRL as M-SLNs and marked on the skin. At times, there were several lymph vessels draining from the injection site, so there were more SLNs than patients. Another 121 nodes were identified by Gd-MRL as distant lymph nodes. During operation, all M-SLNs were easily resected under the guidance of skin marker and 3D MR imaging (Figure 2), and 135 lymph nodes were detected by blue dye as D-SLNs. There was a strong correlation between the numbers of SLNs identified by the two methods (average M-SLNs 1.6 ± 0.6, average D-SLNs 2.25 ± 1.18, Spearman rank correlation coefficient 0.68, *P* < 0.001). Three MRI-detected SLNs were not stained by blue dye.

During surgery, all SLNs identified by either Gd-MRL or the blue dye-guided method were removed, with an average of 2.36 per patient. Twenty-three patients had confirmed metastasis by blue dye-guided method; in 22 of these 23 patients, SLN metastasis was detected by Gd-MRL. Using the blue dye-guided method as the gold standard, the sensitivity of Gd-MRL was 95.65% and the false negative rate was 4.3% for axillary lymphatic metastasis detection.

**Figure 3:**
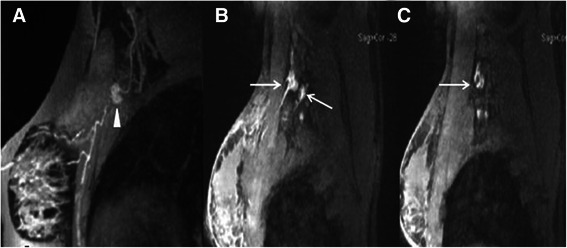
**Comparison of MRL images between benign and malignant SLNs. A:** Benign SLN in a 41-year-old woman with left breast ductal carcinoma. The lymph node displays homogeneous enhancement (white triangle) in Gd-MRL. **B, C:** Malignant SLNs in a 48-year-old woman with left breast ductal carcinoma. Heterogeneous enhancement and enhancement defect were found in Gd-MRL (white arrows).

In Gd-MRL imaging, 28 M-SLNs were confirmed to have metastases; 25 of them showed heterogeneous enhancement and enhancement defect. Using heterogeneous enhancement and enhancement defect as the diagnostic criteria, Gd-MRL gave a sensitivity of 89.29% and specificity of 89.66% in discriminating malignant from benign SLNs (Figure 3). Three of 28 Gd-MRL detected SLNs were confirmed metastatic by pathology, but these were not diagnosed correctly by Gd-MRL due to the small metastasis size (3, 4 and 3 mm). Of the 6 false-positive results, all were attributable to heterogeneous distribution of Gd contrast.

## Discussion

Accurate staging of the axillary lymph node status for breast cancer patients is critical for surgical planning, adjuvant therapy planning, and prognostication. The determination of a negative axillary lymph node status is highly important, as it eliminates the need for the performance of an axillary lymph node dissection, which is well known to be associated with the occurrence of lymphedema, pain, numbness, and range of motion limitations to the shoulder region [20-22]. The sentinel lymph node (s) is the first lymph node or first group of lymph nodes to receive lymphatic drainage from the site of the tumor or the site of injection of the sentinal lymph node localizing agent; if negative, the SLN predicts the status of the remaining distant nodes.

Histopathological examination of sentinel lymph node biopsy is the standard procedure to detect axillary lymph node metastasis. Both radioisotope (technetium-99 m) and blue dyes (isosulfan blue or patent blue) are widely used for lymphatic mapping and SLN identification. The combination of blue dye and radioisotope has a higher SLN identification rate than that of blue dye alone; however, there is no significant difference in the SLN identification rate between blue dye alone versus radioisotope alone [23,24]. However, the radioguided approach to SLN identification requires utilization of radioisotope (technetium-99 m) and gamma detection probe equipment that may not be available at some hospitals without nuclear medicine capabilities. When blue dye alone is utilized for intraoperative SLN identification, the surgeon lacks any specific cues as to the anatomic location of SLNs prior to making the surgical incision. Thus, the lack of being able to visualize the pre-incision anatomical relationship between tumor, lymph vessels, and SLNs when using the blue dye alone approach limits the surgeon’s ability to decide as to where to place the surgical incision. The Gd-MRL approach to SLN identification employs magnetic resonance imaging after interstitial injection (i.e., intradermal periareolar injection) of a conventional gadolinium-based agent. Previously, we developed an effective clinical protocol that can generate high-resolution images of axillary lymphatic vessels and lymph nodes. In our current study, Gd-MRL clearly showed the lymphatic flow from the intradermal periareolar injection site to the axillary region, and resultant identification of the SLNs. The SLNs identified by the Gd-MRL approach correlated well with those SLNs identified by the blue dye alone approach. Using the blue dye alone approach as an acceptable standard of care approach to SLN identification, the sensitivity of the Gd-MRL approach was 95.65% and the false-negative rate was 4.3% for axillary lymph node metastasis detection, indicating that the Gd-MRL approach for breast cancer SLN identification may be clinically feasible and result in an axillary lymph node metastasis detection rate that may be acceptable for use in clinical practice.

In this study, fewer SLNs were detected by Gd-MRL than by the blue dye-guided method. The reason is that in the blue dye-guided method, all dyed nodes were removed as sentinel nodes according to their definition. However, most of them were probably not sentinel nodes, but distant nodes. It is difficult to differentiate these by the standard procedure of sentinel node biopsy using the blue dye-guided method [25]. In contrast, Gd-MRL can accurately discriminate sentinel nodes from distant nodes by visualizing and tracking the lymph flow, which is essential to reduce the false-negative rate. These results may indicate that the accuracy of Gd-MRL is better than that of blue dye-guided methods for SLN identification and has some advantages for SLN biopsy.

Recently, superparamagnetic iron oxide (SPIO)-MR lymphography and iopamidol-CT lymphography with interstitial injection of contrast agent for breast cancer has been reported. Compared with SPIO-MRL, Gd-MRL is more economical and convenient. Superparamagnetic iron oxide is a negative contrast and thus cannot image the lymph vessel. Compared with iopamidol-CT lymphography, Gd-MRL lacks radiation exposure, possibility of anaphylactic shock and nephrotoxic impairment [26,27].

Localization of SLNs in the prone position of MRI differs from that in the operative (supine) position. Several authors have emphasized the importance of preoperative MR imaging in the supine position [28,29]. In the present study, the supine position with elevation of the arms and an MR marking technique using commercially available tablets was adopted for precise preoperative simulation. During surgery, our method was effective, and SLNs could be easily resected under the guidance of skin markers.

In this study, Gd-MRL not only identified SLNs but also diagnosed lymph node metastasis accurately. On a node-by-node basis, using histopathology as the gold standard, Gd-MRL gave a sensitivity of 89.29% and specificity of 89.66%. Previous MR imaging with SPIO-MRL has demonstrated a sensitivity of 84.0% and specificity of 90.9% for the detection of metastasis in SLNs [26]. Our results are consistent with this. Compared with other techniques that have been developed to stage axillary lymphatic node metastases, including dynamic contrast-enhanced MRI and diffusion-weighted imaging techniques, Gd-MRL is more accurate [30-32]. In this study, 3 SLNs with metastases were not diagnosed by Gd-MRL because the metastatic lesion was too small (3, 4 and 3 mm) for the resolution of MRI. Thinner section thickness may improve this.

This study has several limitations. First, there are technical challenges for precise skin marking and SLN correlation, which could not be resolved thoroughly in all MRI studies of axillary lymph nodes [26,27]. What we did was to correlate them based on the following methods: 1) patients were placed in the supine position with their arms elevated, similar to the position used during surgery; 2) using a skin marker, which is usually used for MRI localization; 3) during surgery, each node was removed and correlated with a node on the MR image based on its location. If several nodes lay close to others, they were discriminated by size and morphological character. This is an acceptable and effective method [33]. Second, micrometastasis (<2 mm) was not considered in this study. MR imaging has limited resolution in the present setting and cannot reliably detect micrometastases in lymph nodes. On the other hand, the clinical importance of micrometastases is debatable [34]. Third, Gd-DTPA, a clinically approved intravenous contrast material, was injected in the subareolar breast tissue. Although this off-label use was approved by the institutional review board and all patients provided informed consent, the intradermal toxicity or tolerance of Gd-DTPA needs future investigation. Finally, we only evaluated intradermal periareolar injection. Other possible injection sites, including subareolar, subcutaneous over the primary tumor site, peritumoral, and intratumoral, should be examined in the future.

## Conclusions

In conclusion, we have successfully identified axillary SLNs and detected their metastases in breast cancer patients using magnetic resonance lymphography with a widely available Gd-based contrast agent in a typical clinical setting. The high accuracy as well as the easy protocol suggest a potential value in clinical practice.
